# Prioritization of Variants for Investigation of Genotype-Directed Nutrition in Human Superpopulations

**DOI:** 10.3390/ijms20143516

**Published:** 2019-07-18

**Authors:** Pascal D. Nilsson, Jacklyn M. Newsome, Henry M. Santos, Martin R. Schiller

**Affiliations:** 1 Nevada Institute of Personalized Medicine and School of Life Sciences, University of Nevada Las Vegas, Las Vegas, NV 89154-4004, USA; 2 Food Genes and Me LLC, 929 Via Doccia Court. Henderson, NV 89011, USA

**Keywords:** nutrigenetics, gene-diet interaction, nutrient, superpopulation, SNP

## Abstract

Dietary guidelines recommended by key health agencies are generally designed for a global population. However, ethnicity affects human disease and environment-gene interactions, including nutrient intake. Historically, isolated human populations with different genetic backgrounds have adapted to distinct environments with varying food sources. Ethnicity is relevant to the interaction of food intake with genes and disease susceptibility; yet major health agencies generally do not recommend food and nutrients codified by population genotypes and their frequencies. In this paper, we have consolidated published nutrigenetic variants and examine their frequencies in human superpopulations to prioritize these variants for future investigation of population-specific genotype-directed nutrition. The nutrients consumed by individuals interact with their genome and may alter disease risk. Herein, we searched the literature, designed a data model, and manually curated hundreds of papers. The resulting database houses 101 variants that reached significance (*p* < 0.05), from 35 population studies. Nutrigenetic variants associated with modified nutrient intake have the potential to reduce the risk of colorectal cancer, obesity, metabolic syndrome, type 2 diabetes, and several other diseases. Since many nutrigenetic studies have identified a major variant in some populations, we suggest that superpopulation-specific genotype-directed nutrition modifications be prioritized for future study and evaluation. Genotype-directed nutrition approaches to dietary modification have the potential to reduce disease risk in select human populations.

## 1. Introduction

As genomics holds promise to transform global health and medicine, there is a growing interest in the relationship between genotype and phenotype. Phenotype is derived from both genetic and environmental contributions. The most common environmental influences on phenotype are direct exposure to pathogens and nutrient intake. Herein, we focus on human nutrigenetics. Food-based dietary guidelines are periodically developed by global organizations, especially the Food and Agricultural Organization (FAO) of the United Nations and World Health Organization (WHO). These organizations play a vital role in shaping food policies and guidelines globally, considering unique cultures, food availability, eating habits, food safety, and other factors for each country.

There is mounting evidence that human behavior of diet selection is hereditary. Measurements of heritability separate the genetic and environmental components of any disease or trait. Approximately 60%–80% of the variability in human height is genetically derived with 20%–40% coming from the environment [1]. The heritability is higher in developed countries and lower in developing countries, inferring that the environmental differences are nutrition-based. Consistent with these estimates, the general heritability of food intake patterns is 27%–32% [2]. Other more focused studies impart further support. The heritability of food intake in the Chinese population ranges from 19%–95% depending on types of food and gender [3]. A 12%–24% heritability of eicosapentaenoic acid (EPA) and docosahexaenoic acid (DHA) consumption was identified by a meta-analysis of 17 cohorts [4,5]. The heritability of choosing bread as a food source in Danish and Finnish cohorts ranges from 23%–45% [6].

Once a genetic component of a trait or disease is identified, the next challenge is to decode the genetic variation that explains the heritability, focusing most often on the contributions of individual genes and alleles and more recently on epigenetic changes. This has not yet been well-characterized for human dietary patterns. Very little is known about nutrigenetic variants that affect disease in different populations. Polymorphisms in several genes associated with folate and alcohol consumption, and colon cancer risk have different frequencies in Caucasians and African Americans [7]. There are ethnic effects on plasma polyunsaturated fatty acid levels and preferences for plant protein consumption [8,9]. Although not associated with disease, a principal component analysis identified dietary-driven differences in high abundance plasma proteins among ethnic groups [10]. This study identifies published nutrigenetic variants that have large genotypic differences in different superpopulations [10].

There are new opportunities to explore the role of genetics in the human diet that are supported by strong evidence. The recently reduced cost of sequencing a human genome, exome, microarrays, and genotyping has advanced the understanding of human genetic variation. Variants from >2400 genome-wide association studies (GWAS) consolidated in the GWAS catalog contain over 100,000 disease- or trait-associated variants for rare and common disease with a *p* < 10^−5^ [11,12]. A small number of these variants have clinical utility for disease diagnosis, prognosis, or treatment.

The strong interactions between gene, diet, and disease first became apparent in the 1930s with the identification of phenylketonuria (PKU), a prototypical rare Mendelian disorder characterized by mutations in *PAH* and a deficiency in phenylalanine metabolism [13,14]. In individuals with PKU, the buildup of phenylalanine derived from the diet becomes toxic and lethal [15]. Starting after birth, the major approach to lifelong treatment is a modified diet with low levels of phenylalanine [16]. This clinical nutrition therapy is similar to other nutrition therapies for the treatment of galactosemia and hereditary fructose intolerance [17,18].

Over the past two decades, advances in GWASs and studies with focused candidate gene panels have enabled scientists to more rapidly identify genetic variants in gene-diet interactions and their associations with disease. This new capability has driven the emergence of the field of nutrigenetics [19]. Nutrigenetics assesses how a person responds to specific nutrients based on the variation within their genome. Many studies and trials have identified nutrigenetic variants associated with common diseases such as colorectal cancer, obesity, type 2 diabetes, and cardiovascular disease to name a few [20,21,22,23].

**Figure 1:**
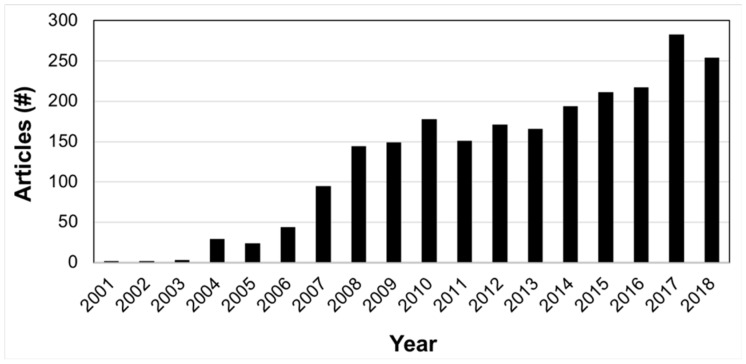
Bar graph of the nutrigenetics publication trend. The bar graph shows the number of nutrigenetic publications per year, beginning in 2001. The total number of papers is 2,317. Abstracts were identified by querying PubMed, with terms related to nutrigenetics and disease. Examples are indicated in the Materials and Methods section.

The number of publications in nutrigenetics has steadily increased over the past 17 years (Figure 1). Despite a significant amount of research in this field, the data have not been systematized into an accepted model to assess a person’s genetics acquired by panel genotyping, single nucleotide polymorphism (SNP) microarrays, whole-exome sequencing, or whole-genome sequencing. The nutrigenetics.net database is a public collection of nutrigenetics literature. Most of the SNPs related to nutrigenetics are not present in other structured databases such as ClinVar, LOVD, and HGMD. These structured databases hold limited nutrigenetic data, but house clinical SNPs, disease-associated SNPs, and human genetic variation data related to traits and behavior [24,25,26,27].

Several nutrigenetic companies have emerged with goals of prescribing food intake and exercise with genetics. These companies offer clients genotyping, and/or secondary data analysis. The results are then processed to suggest personalized nutritional modification strategies. However, this new field is not without controversy with some criticism of nutrigenetic testing companies [28,29,30]. However, others have pointed out that some of the criticisms are not factually based and are potentially damaging to private interests [31,32]. Thus, the challenge remains to improve upon and disclose the value of genetic testing procedures based on quality scientific evidence.

The main goal of this study is to prioritize SNPs for validation of genotype-directed nutrition dietary modifications to reduce the burden of disease risk in healthy people based upon genetic variant frequencies in global superpopulations. Genotype-directed nutrition was previously defined and the five major superpopulations are as defined in the 1000 genomes project [33,34]. Herein, the term “genotype-directed nutrition” reflects dietary modifications based upon common nutrigenetic variants with high frequencies in at least one human population. This is in contrast to personalized nutrition, where the diet is precisely designed for each individual and not a population. The proposed population stratification and genotype-directed nutrition could be further tested and if validated provide significant health benefits. We do not know of another report that globally prioritizes nutrigenetic studies for validation studies based upon SNP frequency. Note, that this effort is focused on diets for normal people, and not for clinical nutrient therapies, which are medical treatments and should be subject to stricter clinical validation studies.

## 2. Results

### 2.1. Literature Annotation for Nutrigenetic Database

**Figure 2:**
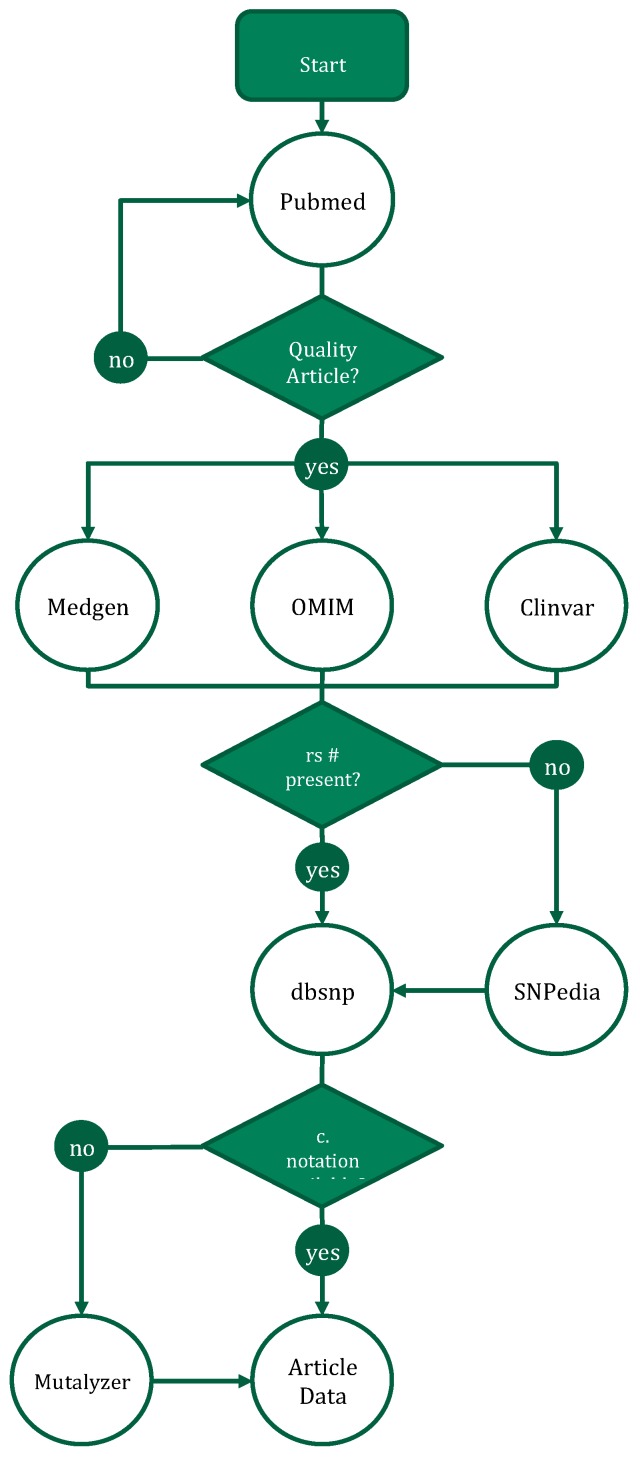
Nutrigenetic paper annotation workflow.

The first step needed for creating genetic-driven nutrition modification was to create a nutrigenetic database extracting information from published articles into structured data. There were ~2,300 nutrigenetics articles published since 2001 (Figure 1). A database was built from published studies employing well-defined criteria for variant selection following the process schema diagram in Figure 2.

We evaluated each article for quality based on reported odds-ratios and *p*-values and optional confidence intervals. Any nutrigenetics study reporting a *p*-value (*p* < 0.05) was considered for further annotation [35]. Any GWAS was considered if it met the *p* < 5 × 10^−8^ threshold for genome-wide significance, which incorporates the Bonferroni correction for multiple testing. An annotation entry also required reported log odds ratio (0.97 < OR > 1.03) for an association between a nutrient and a disease or endophenotype outcome. We selected articles that had a log-odds ratio for the disease or endophenotype and also for the effect of the nutrient intake and variant on the disease risk. Protective or risk factor variants had odds ratios for gene-diet interactions greater than that of the nutrigenetic population and vice-versa for a protective variant. Since confidence intervals are not always represented in articles, and while useful, they are not essential to the annotation process, thus can be entered as null in the database.

Articles meeting these criteria were next cross-referenced with MedGen, Online Mendelian Inheritance of Man (OMIM), and ClinVar. If the variant did not have a dbSNP identifier, one was obtained from SNPedia. If C. notation for the variant was not available, the information was obtained with Mutalyzer. The PubMed identifier was also recorded.

### 2.2. Summary Statistics for Nutrigenetic Variants

The current database version has 156 gene-diet interactions with 101 unique variants in 84 genes passing the annotation quality criteria (Table 1). These variants account 145 nutrient intake or dietary suggestions, resulting in 290 total entries. Multiple entries can come from a study due to multiple variants reaching significance or multiple diet groups accompanying a particular variant within the same study. This set of variants accounted for the risk of 38 phenotypes, most of which were diseases, although some constitute endophenotypes. The ORs ranged from 0.07 to 35 with most SNPs (67%) increasing risk for the phenotype.

An example disease, colorectal cancer, is associated with 23 unique variants and affected by 34 different gene-nutrient interactions. The majority of the variants correlate with an increased risk of disease, which can be mitigated by nutrient intake. Folate was consistently identified in many gene-diet interactions, increasing or lowering disease risk dependent upon the amount ingested. A cohort study examining colorectal cancer identified gene-diet interaction between the *MTHFR* gene and folate intake in Koreans. Carriers of the C base in the C677T SNP variant had a decreased risk with high intake of folate (>282 μg/d: OR 0.62, *p* < 0.002, confidence interval [CI] 95%: 0.46–0.84) [36]. This is just one example disease where evidenced-based modifications to a person’s diet could ameliorate the increased risk of disease [36].

### 2.3. Genotype-Directed Nutrition Prioritization for Superpopulations Based on Nutrigenetic Variants

**Figure 3:**
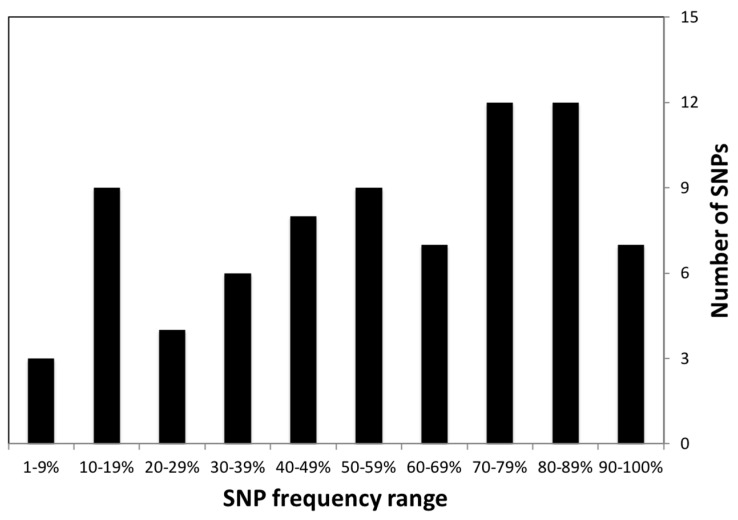
Bar chart with the number of annotated nutrigenetic variants vs. the frequency range. Variant data for superpopulation frequency ranges is from the phase III release of the 1000 Genomes Project [37].

Studies of gene–diet interactions are designed to generally identify common variants in the population that have detectable effects from prevalent SNPs. Thus, it is no surprise that our nutrigenetics database has 37% of nutrigenetic SNPs with global SNP frequencies for nutrigenetic variants >50%, with all variants having a wide range of frequencies (Figure 3). Furthermore, of the 101 unique variants, six had global SNP frequencies >90%. We reasoned that these variants could be utilized to improve population health. Therefore, we further examined how SNP frequencies of these variants varied among superpopulations (Africans, Americans, East Asians, Europeans, and South Asians) by analyzing the 1,000 genomes phase 3 data. There were 17 SNPs where comparison of two superpopulation had an F_ST_ > 0.5, with values ranging up to 0.71.

An example in Table 2 shows that a variant in the *VDR* gene is much less frequent in Africans with the F_ST_ for all superpopulations ranging from 0.06 to 0.71. We, therefore, examined those variants with SNP frequencies of above 50% in at least one superpopulation (Table 2).

Studies on these SNPs suggest nutritional changes for the superpopulations that may have a population-wide benefit for the diseases associated with these variants. For all populations, low fat, high n-3 PUFA, low n–6 fatty acids, and high folate may reduce the global disease risk for five common disorders (Table 3). While high n–6 fatty acids are a general suggestion, people with this genetic variant might consider low n–6 fatty acids in their diets [38]. There are also several superpopulation specific suggestions. For example, low calcium (<680 mg/day), low alcohol and high Vitamin D consumption for East Asians may be a dietary means to reduce population incidence of prostate cancer and obesity. This may require stratification by subgroups as addressed in the discussion section.

## 3. Discussion

The human condition is not just affected by genes, as many traits and conditions are also affected by physical location, environmental exposure, exercise, microbial interactions, and diet. Two prospects are information and genetic-driven, personalized medicine and prevention. While there are several approaches to prevention, in this article, we focus on genetic-driven food and nutrient intake as a source of prevention in the emerging field of nutrigenetics. Food is an important daily exposure factor that provides a wide variety of nutrients, non-nutrients, and even chemical contaminants that can modulate disease risks. For most human diseases, the risk is a combination of heritability, environmental factors from nutrient intake, and gene-diet interactions.

With the recent advances in commercialization of recreational genetics and further development of genetic investigations, there is a recent re-emergence of nutrigenetics companies. We considered the qualities of implementing scalable nutrigenetics that are needed for successful implementation. In this paper, we expand on three that we think are important, variant quality, collection of nutrigenetic data into a structured database, and how this information can be leveraged to prioritize studies for population-specific diets.

### 3.1. Variant Quality

Variant quality is a concern for several reasons that became more apparent as we annotated variants from the literature. Upon completion of our variant annotation database, the Global Nutrigenetics Knowledge Network (GNKN) reported standards for the quality and utility of nutrigenetic variants [29]. Many nutrigenetic research studies do not reach the rigor of the draft of standards proposed by the GNKN. Therefore, we revised the goal of this study to compile and analyze existing nutrigenetic studies to prioritize variants for validation studies. We had independently used a *p* < 0.05 corrected for multiple testing criteria, a log odds ratio with a significant change, and confidence intervals.

There are several guidelines (STEGA, EGAPP, and GRADE) for clinical validity and utility of genetic tests [29,39,40,41]. For the current state of knowledge and difficulties in nutrigenetic studies, the clinical stringent criteria are generally too strict. They are designed for medical care, where errors in treatment could have life-threatening or life-altering effects. We emphasize that the purpose of our genotype-directed nutrition study is to prioritize variants to reduce disease susceptibility risk of healthy people with preventative or early detection strategies and not for clinical medicine.

Some additional GNKN guidelines will be useful for classification of nutrigenetic variants. In particular, our variants should be further characterized for the hierarchical level of evidence supporting the variant marker, as well as the magnitude of effect as suggested by EGAPP [39]. The European Food Safety Authority (EFSA) scientific validity guideline is based on a number of studies, rather than a statistical outcome from a meta-analysis, which has a stronger scientific rationale [29]. The framework does not include a model to classify more complex metrics of genetic architecture such as genetic and polygenic risk scores. These metrics are geared toward susceptibility, which in general explain more of the heritability for common traits, conditions, and behaviors such as nutrient intake. These metrics are better suited to evaluate risk and are becoming rapidly accepted as approaches of choice for susceptibility to common disease [42,43,44].

These criteria fit that of a rare variant, rather than a common variant. Given the generally low number of participants in nutrigenetics studies when compared to large clinical trials, it is difficult to identify rare variants that have large effects as is the case for Mendelian disorders. In fact, this is the claim of the blood group diet where there are alleles of large effect, but these blood type alleles are not rare. Nevertheless, rare variants are of limited utility for genotype-directed nutrition in large populations unless many are identified. Currently, there are only a few examples reported for Mendelian disorders, as exemplified earlier by mutations in the *PAH* gene and clinical treatment of patients afflicted with PKU by dietary therapy [16,45].

In general, the 1,000s of new GWAS studies over the past decade support a model for a common disease where there are many common variants of small effect sizes, perhaps triggered by a specific combination with a rare variant. A more common recent practice is to sum the smaller risk of these large sets of common variants associated with any affliction into a genetic or polygenic risk score. This better models the polygenic and heterotypic nature of common disease. The effect of food intake on common disease is more likely to resemble a common disease, thus may be better approached by genetic risk scores. However, the field of nutrigenetics is yet to adopt this approach. This is a limitation that will need to be addressed in the future and modeled in current guidelines.

### 3.2. Nutrigenetics Database

Nevertheless, within the context of these limitations, we sought a way in which we could take advantage of the growing number of nutrigenetics studies. There are well-developed nutrient databases, but no comprehensive nutrigenetics databases [46]. We developed a data model to capture critical nutrigenetic information and annotated ~156 gene-diet interactions from a comprehensive review of the nutrigenetics literature. While all studies were statistically significant, we recognize that for most of them, the study size is of small by today’s standards, may not have been repeated, and therefore, some results may not repeat in a larger study or a meta-analysis. Furthermore, compliance with nutrient intake may be challenging in these studies. One particular challenge was modeling ethnicity, epistasis and the broader applicability of variants identified in a study focused on a specific ethnic population. Nevertheless, the value of our nutrigenetics database is synergistic with other nutrition-related databases that are publicly available such as the U.S. Department of Agriculture (USDA) nutrient database and PhenolExplorer for phenol content in foods [47].

### 3.3. Genotype-Directed Nutrition for Populations

Many genetic studies focus on specific ethnic populations, or pedigrees to isolate variants from variable genetic backgrounds. Even though ~100 nutrigenetic variants met our quality stringency criteria for annotation, the knowledge gained from these genetic studies is not yet used in the design of population-specific diets. To explore whether this information could potentially be useful for adjusting diet design, we examined F_ST_ values for human populations. A small group of the SNPs were common in all populations, while a larger subset had significant changes in SNP frequencies between populations. Since the gene-diet interaction SNP was the major SNP in one or more superpopulation this information could be used to suggest superpopulation-specific nutritional changes when compared to a recommended diet. The summation of this approach yielded a collection of nutrition suggestions for each superpopulation, which can be further tested for validation. Other aspects of nutrigenetics, such as ethics, food supply, and food safety would also need to be considered.

### 3.4. Limitations

While the potential of a nutrigenetic precision diet is an attractive and intuitive concept in the prevention and management of chronic diseases, there are several limitations that must be considered in the interpretation of these data.

We recognize that variant interpretation may be more complex as the gene-diet interactions may be more polygenic in nature, like common diseases. In this case, a genetic risk score may be more suitable than SNPs associations. Indeed, the problematic nature of using single SNPs to predict complex traits are well known [48]. Most current nutrigenetics studies are limited examining small population sizes and a portion of these studies may not repeat upon a more rigorous design. However, we are using these studies for prevention and not for clinical intervention, thus the bar for quality does not have to be as high. Nevertheless, ideally, validation studies on larger populations should be tested prior to implementing a nutrigenetic recommendation. There are also instances where different nutrigenetic variants may influence the same phenotype and dietary associations [22,49]. However, genetic risk may be better predicted at the group level, as suggested in this paper, rather than for individualized predictions [48].

The majority of nutrigenetic studies differ in study design, population demographics, and sample size, thus introducing bias. The SNPs identified from one ethnic group may not be more broadly generalizable to other populations due to epistasis. Many of these studies must be replicated in other population types since genetic variation across ancestries and geographical regions exist [50]. Furthermore, with the recent ease and frequency of human migration, the superpopulation dietary suggestions are based on SNP frequency, which is not applicable to ‘interracial’ populations. Potential solutions are to derive dietary suggestions from an individual superpopulation(s) of ancestral origin or to analyze an individual’s genetic file.

Another limitation in the current field of nutrigenetics is research factors in the design of studies that identify variants. This includes the type of study design (e.g., meta-analysis, randomized trial, longitudinal, prospective, cross-sectional). Other characteristics such as outcome, effect size, population size, control groups, and confirmation by a separate research study should also be considered. Furthermore, conclusions drawn from a study with “significant” *p*-values are under scientific scrutiny and should also include effect sizes, Bayesian measures, and reproducibility with meta-analyses from multiple studies [51]. Certainly, our database is limited by the qualities of available studies and that is why we suggest that the data we collected and modeled be used primarily to prioritize hypotheses for future investigation.

Population-level dietary recommendation is standard practice and we propose that genotype-directed nutrition for genotypes with high frequencies in populations could provide significant health care savings and reduce morbidity and loss of productivity. However, another key consideration is that some nutrient suggestion may need to be codified for specific groups within the population. For example, nutrigenetic variant suggesting low calcium intake (<680 mg/dL) for healthy adults needs to be carefully considered for specific population groups such as infants, adolescents, pregnant women, postmenopausal women and the elder population where this suggestion may not be applicable or even harmful.

## 4. Material and Methods

### 4.1. Data Sources for Nutrigenetic Variants

The majority of nutrigenetics knowledge exists in free form text of peer-reviewed publications. Due to current limitations in interpretation and mining information out of the free-form text, we decided to adopt manual annotation, which, while more time consuming, has the advantage of better accuracy. We reviewed the current scientific literature regarding dietary nutrients and nutrigenetic variants.

The PubMed database was queried with keywords and phrases to collect relevant nutrigenetic articles. Examples of such query phrases included, but were not limited to, “nutrigenetic”, “gene-diet interaction”, “diet intake”, “polymorphism”, “consumption”, and “nutrient intake and gene-diet interaction”. We also searched for combinations of these keywords and queries where “diet” was replaced with “food source”, “nutrient” or “mineral”. These search queries required multiple variations to find the relevant articles for annotation. A second major source of nutrigenetic variants was the GWAS catalog [11,12].

Information in the primary literature or GWAS catalog was extracted into a nutrigenetic data model (see Results). Variants were cross-referenced to National Center for Biotechnology Information (NCBI) data such as the PubMed, Entrez Gene, and MedGen databases where applicable. Entrez Gene and GeneCards were sources of gene summaries. Diseases were referenced with OMIM. dbSNP provides SNP IDs for each variant, and for cross-reference with several other databases such as the 1000 Genomes Project.

All variants were entered with HGVS notation. For the c, p, and g fields, c. is the nucleotide position in the gene, p. is the position of the amino acid substitution in the gene, and g. is the nucleotide position within the entire chromosome. The reference genome for the database is GRCh37.p13. Although there are newer reference genomes, data that were included from several other databases and websites were referenced against GRCh37.p13. The USDA nutrient database provided a source of nutrients in foods [46].

### 4.2. Population Frequencies and F_ST_ Values of Nutrigenetic Variants

The frequencies for each SNP variant were retrieved from the 1000 Genomes Project, phase 3 browser running Ensembl version 80, and referenced against GRCh37. The 1000 Genomes Project, phase 3 utilizes more than 80 M short variants with genotypes of 2504 individuals across 26 global populations [37]. The human superpopulations are all, African (AFR), Admixed American (AMR), East Asian (EAS), European (EUR), South Asian (SAS). SNP frequency data for human superpopulations were from the 1000 genomes project. The fixation index (F_ST_) values for pairs of superpopulations were calculated from SNP frequency data with Excel. F_ST_ is calculated from the equation: F_ST_ = σ^2^_S_/σ^2^_T_ where σ^2^_S_ is the variance in the subpopulation and σ^2^_T_ is the variance in the total population. These values measure the differences in frequencies of the SNPs across subpopulations. 

### 4.3. Data Model

**Figure 4:**
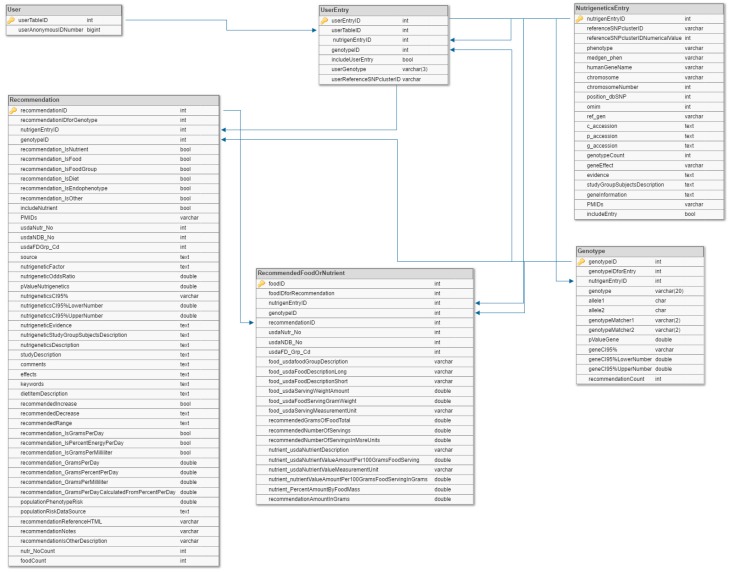
SQL schema for the nutrigenetic-nutrient database. Table names are in the dark grey header of each table. Tables contain fields with corresponding data types. The primary and foreign keys connecting tables are indicated by lines with arrows.

Nutrigenetics variant data annotated from the literature, NCBI databases, and the USDA nutrient data was modeled in a MySQL database. The database has six tables (Figure 4). These include a user table (“User”) (with an anonymized user ID), a table linking each user to the relevant nutrigenetic variant entries (“UserEntry”), the genetic variant table (“NutrigeneticsEntry”), a genotype table (“Genotype”), a dietary suggestion table, and a table with foods and nutrients relevant to the entries on the suggestion table (“FoodOrNutrient”). The “NutrigeneticsEntry” table contains general information about the database variants with gene summaries, the dbSNP ID, phenotype, and chromosomal position. Each entry in the “NutrigeneticsEntry” table corresponds to one or more entries in the “Genotype” table. These entries include information on the effected minor frequency SNPs, including SNPs and *p*-values, odds ratios, and confidence intervals for the variant-disease interaction.

Each entry in the “Genotype” table is associated with entries in the “foods and nutrients” table. This table contains information on each dietary suggestion, such as the suggestion type (which may be to consume a certain portion of a food, nutrient, or food group, or to monitor a particular endophenotype), PubMed ID, study description, population risk data, and the *p*-value, OR, and CIs for the variant-phenotype-diet interaction for the suggestion. Each “foods and nutrients” entry is, in turn, associated with multiple entries on the “FoodOrNutrient” table. This table contains the relevant USDA nutrient database information for the suggested foods, such as the number of servings needed to provide the suggested daily value of a nutrient and the food’s nutrient content.

## 5. Conclusions

1. Nutrigenetic variants with high superpopulation frequencies can be used to prioritize dietary modifications for the purpose of reducing disease risk for human superpopulations with the potential for widespread health benefits.
2. The proposed superpopulation genotype-directed nutrition modifications will need to be validated in a research study.

## 6. Data Availability

The database is proprietary and licensed to a company, but may be used for research purposes. The database is available for collaboration upon request or for distribution through a license agreement.

## Figures and Tables

**Table 1:** Statistics for construction of the nutrigenetic database.

Category	^2^Number
Articles	67
Annotations	156
Phenotypes	36
Genes	84
^1^SNPs	101
Protective	52
Risk	104
^1^OR range (Avg)	0.07–35 (2.17)
P-value range (Avg)	3.5 × 10^−5^ – 0.05 (0.018)
Diet types	106
Participants range (Avg)	1–16,624 (1106)

*Note:*^1^ Abbreviations are: SNP = single nucleotide polymorphism; OR = Odds ratio; Avg = average; ^2^ Numbers in parentheses are averages.

**Table 2:** Frequencies for select SNPs with >50% population difference.

^2^dbSNP ID	Gene	Disease	Dietary Change	^1,2,3^Superpopulation SNP Frequency					
				ALL	AFR	AMR	EAS	EUR	SAS
rs9997745	*ACSL1*	Metabolic Syndrome	^1^Low-fat (<35% energy), high-PUFA diet (>5.5% energy)	78	40	87	100	85	93
rs6008259	*PPARA*	Hypercholesterolemia	Low n–6 fatty Acid (≤7.99 g/day)	73	86	24	100	82	92
rs6087990	*DNMT3B*	Colorectal Cancer	^1,4^High RBC folate	68	76	63	92	37	68
rs3790433	*LEPR*	Metabolic Syndrome	^1,5^Low n-6 PUFA, high n-3 PUFA	59	23	67	84	77	58
rs11568820	*VDR*	Prostate Cancer	Low calcium (<680 mg/day)	54	11	82	60	77	64
rs512535	*APOB*	Metabolic Syndrome	Low fat (<35% energy)	53	19	51	81	51	73
rs10495563	*ADAM17*	Obesity	^6^Low n-6 fatty Acid	52	30	56	90	34	58
rs2287161	*CRY1*	Metabolic Syndrome	Low carbohydrate (% of energy intake <41.7%)	46	64	52	13	45	54
rs3827730	*FAF1*	Alcohol Dependence	^7^Low amounts of alcohol	38	7	52	79	35	28
rs2424913	*DNMT3B*	Adenoma, Colorectal Cancer	No alcohol	31	33	36	1	59	29
rs1801181	*CBS*	Colorectal Cancer	^1,4^High RBC folate	30	2	19	57	39	36
rs2424909	*DNMT3B*	Colorectal Cancer	Moderate alcohol >0 and <1.7 drinks/week	28	8	36	8	63	31
rs1378942	*CSK*	Hypertension	^1^1.8 g/day of EPA and DHA	24	3	33	18	61	16
rs2168784	(Intergenic)	Alcohol dependence	no alcoholic drinks/week	24	62	10	9	10	13
rs1229984	*ADH1B*	Alcohol dependence	no alcoholic drinks/week	16	0	6	70	3	2
rs75038630	*NADSYN1*	Abnormal Eating Behavior	High vitamin D (>75 nmol/L)	2	0	4	100	6	3

*Note:*^1^ Abbreviations are as in Table 1 and: PUFA = polyunsaturated fatty acid; RBC = red blood cell; EPA = eicosapentaenoic acid; DHA = docosahexaenoic acid; g= gram; mg = milligram; L = liter; population abbreviations are defined in text. ^2^ SNPs with FST > 0.5 for two superpopulations. ^3^ SNPs with a >50% frequency are shaded gray. ^4^ Low levels of RBC folate is defined as (<484 ng/mL) and associated with a risk, therefore, high levels of folate consumption should offset this risk and are reported as high RBC folate. ^5^ Low PUFA status (<45.85% of total measured fatty acids) if the diet is low (less than the median) plasma n-3 and high (n-6) PUFA. ^6^ Undefined amount in the article. ^7^ Dietary change: non-alcohol dependence or low occurrence of drinking alcohol.

**Table 3:** Nutrigenetic dietary suggestions for superpopulations.

Category	^1^Diseases	^1,2^Dietary Suggestion
All	Metabolic Syndrome, Hypercholesterolemia, Colorectal Cancer, Prostate Cancer, Obesity	Low-fat (<35% energy), High-PUFA diet (>5.5% energy), Low n–6 Fatty Acid (≤7.99 g/day), Low Calcium (<680 mg/day)
AFR	Hypercholesterolemia, Alcohol dependence	Low n–6 Fatty Acid (≤7.99 g/day), 0 alcoholic drinks/week
AMR	Colorectal Cancer, Prostate Cancer, Obesity, Alcohol Dependence	High PUFA, Low Calcium (<680 mg/day), ^3^Low n-6 Fatty Acid
EAS	Hypercholesterolemia, Prostate Cancer, Obesity, Alcohol Dependence, Abnormal Eating Behavior	Low n–6 Fatty Acid (≤7.99 g/day), Low Calcium (<680 mg/day), ^3^Low n-6 Fatty Acid, High vitamin D (>75 nmol/L)
EUR	Hypercholesterolemia, Prostate Cancer, Adenoma, Hypertension	Low n–6 Fatty Acid (≤7.99 g/day), Low Calcium (<680 mg/day), 1.8 g/day of EPA and DHA
SAS	Hypercholesterolemia, Prostate Cancer, Obesity	Low n–6 Fatty Acid (≤7.99 g/day), Low Calcium (<680 mg/day), ^3^Low n-6 Fatty Acid

*Note:*^1^Table 3 is a summary of information from Table 2. ^2^ Abbreviations are as in Table 1 and Table 2. ^3^ Undefined amount in the article.
